# *Kocuria flava*, a Bacterial Endophyte of the Marine Macroalga *Bryopsis plumosa*, Emits 8-Nonenoic Acid Which Inhibits the Aquaculture Pathogen *Saprolegnia parasitica*

**DOI:** 10.3390/md21090476

**Published:** 2023-08-29

**Authors:** Ynon Deutsch, Mohamed Samara, Ahmed Nasser, Ilana Berman-Frank, David Ezra

**Affiliations:** 1 Department of Plant Pathology and Weed Research, The Volcani Institute, Agricultural Research Organization, Rishon LeZion 7528809, Israel; ynond@volcani.agri.gov.il; 2 Leon H. Charney School of Marine Sciences, Department of Marine Biology, University of Haifa, Haifa 3103301, Israel; iberman2@univ.haifa.ac.il; 3 The Inter-Institutional Analytical Instrumentation Unit (IU), The Volcani Institute, Agricultural Research Organization, Rishon LeZion 7528809, Israel; mohamed@volcani.agri.gov.il (M.S.); nasser@volcani.agri.gov.il (A.N.)

**Keywords:** endophyte, macroalga, secondary metabolite, 8-nonenoic acid, aquaculture, disease management

## Abstract

Secondary metabolites—organic compounds that are often bioactive—produced by endophytes, among others, provide a selective advantage by increasing the organism’s survivability. Secondary metabolites mediate the symbiotic relationship between endophytes and their host, potentially providing the host with tolerance to, and protection against biotic and abiotic stressors. Secondary metabolites can be secreted as a dissolved substance or emitted as a volatile. In a previous study, we isolated bioactive endophytes from several macroalgae and tested them in vitro for their ability to inhibit major disease-causing pathogens of aquatic animals in the aquaculture industry. One endophyte (isolate Abp5, *K. flava*) inhibited and killed, in vitro, the pathogen *Saprolegnia parasitica*, an oomycete that causes saprolegniasis—a disease affecting a wide range of aquatic animals. Here, using analytical chemistry tools, we found that Abp5 produces the volatile organic compound (VOC) 8-nonenoic acid. Once we confirmed the production of this compound by the endophyte, we tested the compound’s ability to treat *S. parasitica* in in vitro and in vivo bioassays. In the latter, we found that 5 mg/L of the compound improves the survival of larvae challenged with *S. parasitica* by 54.5%. Our isolation and characterization of the VOC emitted by the endophytic *K. flava* establish the groundwork for future studies of endophytic biocontrol agents from macroalgae. Use of this compound could enable managing oomycete agricultural pathogens in general, and *S. parasitica* in particular, a major causal agent in aquaculture diseases.

## 1. Introduction

Endophytes are microorganisms, mostly fungi and bacteria that inhabit the inner plant tissue; they can be found in all plants in nature [1]. They form a mutualistic relationship with the plant host which provides the endophyte with a living niche and food, while the endophyte provides tolerance to, and protection against biotic and abiotic stressors. Endophytes have been found to secrete specialized metabolites that contribute to their survival in hostile environments [2,3], termed secondary metabolites. Secondary metabolites can be categorized into various chemical groups: alkaloids, flavonoids, phenolic acids, fatty acids, quinones, steroids, alcohols, tannins, terpenoids, ketones, xanthones, and many others [4]. Endophytes use their secondary metabolites as antifungals, antibacterial, antivirals and antioxidants [5,6,7], as part of their defense against competitors. As a side effect, those metabolites benefit the host plant by protecting them against pests and other stressors. Some of those secondary metabolites are also known for their pharmaceutical properties (i.e., as anticarcinogens, anti-inflammatories, antiarthritics, antidiabetics, and immunosuppressants [4,8]).

Endophytes comprise many phyla. For example, Actinobacteria—gram-positive bacteria, many of which are producers of medicinal antibiotics—can be found in terrestrial and aquatic environments [5,9,10,11]. One such actinomycete is *K. flava*, first described by Zhou et al. (2008) as yellow, aerobic, non-motile and coccoid cells. It can be found in many environments, such as air [12], soil, plants [13,14], roots [15], marine sediment [16], sponges [17] and algae [18,19]. *K. flava* is known for its versatile abilities, showing methanol catalytic activity, thermotolerance, antifouling activity, chromate tolerance, plant growth induction and more [15,19,20]. We previously isolated *K. flava,* isolate ABp5, as an endophyte from the marine macroalga *Bryopsis plumosa* [18]. *B. plumosa* is a seaweed of the phylum Chlorophyta. *Bryopsis* sp. is known for their ability to attract, select, and maintain specific endophytes within their cells; they use these endophytes for wound repair and propagation in the face of biotic stresses [21]. 

In our previous study, we isolated seven different endophytes from *B. plumosa*, all of them were found to be biologically active against aquaculture pathogens. *K. flava* isolate ABp5 was active against *S. parasitica* in in vitro assays [18]. *S. parasitica* is an oomycete (water mold) causing saprolegniasis, a disease affecting freshwater fish eggs and juvenile fish in hatcheries worldwide. In mature fish, it is characterized by visible white or gray patches of filamentous mycelium on the body or fins. *Saprolegnia* infections were previously controlled with Malachite green, but since this compound was banned internationally in 2002, this oomycete has become a serious problem in the aquaculture industry, mainly of catfish (*Ictalurus* spp.), salmon (*Salmo* spp.), and trout species [22,23]. 

Aquaculture is the fastest growing food industry. The increasing demand for healthy animal-sourced protein and a rapidly growing economy have resulted in an annual growth rate of 5.8% for the aquaculture industry since 2001 [24]. The salmon farming industry has grown exponentially in the last three decades, with Norway and Chile as the major suppliers. This industry holds a 4.5% of the world’s aquaculture industry with fish yield of 2.5 million tons in 2018. It has become an integral part of the global aquaculture market due to its large economic share. It is estimated that 10% of all hatched salmon die due to *Saprolegnia* infections, causing major financial losses for this industry [23,25,26]. 

In this study, we isolated and identified an active compound from the endophytic bacterium *K. flava*. The compound was tested for bioactivity against the aquaculture pathogen *S. parasitica* by both in vitro and in vivo bioassays, demonstrating potential for using as a control agent for one of the major diseases in the world aquaculture industry today.

## 2. Results

### 2.1. Bioactivity Assays

**Figure 1:**
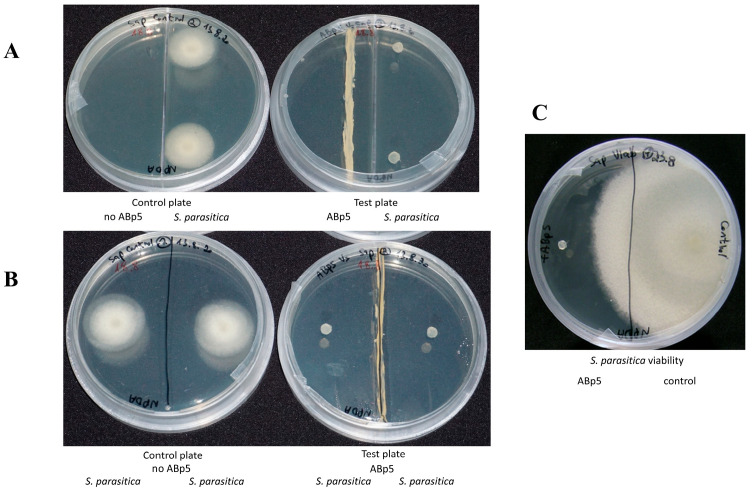
In vitro bioactivity assays of *K. flava* isolate ABp5 against the aquaculture pathogen *Saprolegnia parasitica*. ABp5 was grown in the middle of an NPDA plate for a week (A + B, right plates) before a plug of solid medium harboring live *S. parasitica* mycelia was added to the plates. Control plates were inoculated with *S. parasitica* in the absence of ABp5 (A + B, left plates). (**A**) A two-compartment petri plate used for the bioactive volatile emission assay. The divider prevents any direct contact of ABp5 and its secreted secondary metabolites with the pathogen, allowing only volatile movement in the headspace between the compartments. (**B**) One-compartment petri plate. In this assay, ABp5 can inhibit the pathogen through both direct and indirect contact with secondary metabolites (secreted or emitted through the agar or the headspace, respectively). In both panels A and B, *S. parasitica* was fully inhibited by ABp5. (**C**) Pathogen viability assay. *S. parasitica* from both assays (direct and headspace exposure) were transferred to a plate with new medium plate in the absence of ABp5. There was no growth of pathogens from either assay (shown for the headspace experiment only); a plug from the control plate was viable and grew as expected (right side of the plate).

The endophytic isolate ABp5 fully inhibited *S. parasitica* in an antibiosis test performed on synthetic solid growth medium in a closed petri dish (Figure 1B). This activity was not obtained when we cultured the isolate in liquid medium followed by liquid/liquid chloroform crude extraction of the secreted secondary metabolites. We therefore assumed that the active metabolites are volatiles and not secreted into the media. To examine this assumption, we performed an assay designed to evaluate volatile activity, using a two-compartment petri dish that allows the volatiles emitted by the bacteria, but not the secreted metabolites, to migrate into the oomycete chamber. Full inhibition of the pathogen was observed, restoring the endophyte activity that we had observed in the solid medium bioassays (Figure 1A). *S. parasitica* plugs from both the solid antibiosis and volatile two-compartment assays were examined for viability. In both cases, the pathogens transferred onto a plate with fresh medium without the endophyte, were dead (Figure 1C). 

### 2.2. VOC Identification

To further understand the basis of ABp5′s bioactivity, we chemically analyzed the gas phase of the endophyte grown on NPDA agar slant by GC–MS. Table 1 presents the 10 different suggested compounds identified by GC–MS (see Table S1 for additional information). These compounds belonged to the ketone, alcohol, phenol, fatty acid and ester families. Nine of them were commercially available (oxime-, methoxy-phenyl-_ was not available).

#### 2.2.1. Testing Standards for Bioactivity against *S. parasitica*

**Figure 2:**
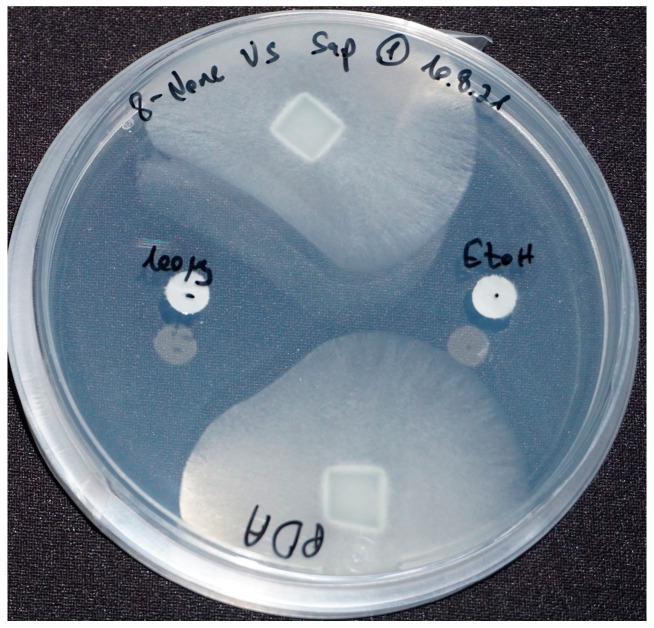
8-nonenoic acid inhibitory activity against *Saprolegnia parasitica* in agar plates exam. Inhibition was recorded after seven days. *S. parasitica* plugs, 100 μg of 8-nonenoic acid (left disk) and ethanol (right disk) as a control. Inhibition is expressed as avoidance of growth towards the paper disk loaded with 8-nonenoic acid while the *Saprolegnia* mycelium grows with no disturbance towards the ethanol loaded discs.

All nine purchased compounds were examined for their ability to inhibit the growth of *S. parasitica*. 8-nonenoic acid (Figure 2, on the left) was the only to demonstrate strong growth inhibition, with 100 μg of 8-nonenoic acid causing an inhibition zone of 17–21 mm on agar plates. Ethanol as a control did not cause inhibition, moreover it can be seen that the oomycote is growing towards it (Figure 2 on the right). 

#### 2.2.2. 8-Nonenoic Acid Identification by GC Comparison 

**Figure 3:**
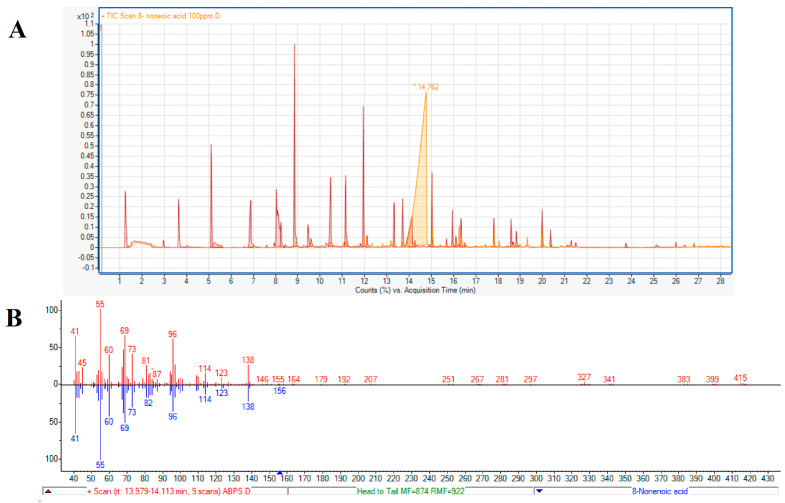
Identification of 8-nonenoic acid. (**A**) Comparative analysis of GC–MS retention times (RT) of ABp5 and authentic standard. 8-Nonenoic acid standard peak (light orange) with RT of ~14 min post-injection and the ABp5 peaks (red), released from the SPME, showing one peak at ~14 min post-injection with similar features (shape and slope) as the authentic standard. (**B**) Fractionation comparing the peak identified as 8-nonenoic acid secreted by ABp5 (red-line columns above axis) and the peak produced by the purchased authentic standard (blue-line columns below axis). The fractionations given by the GC–MS are identical for both.

A second method to test the identity of the active compound as 8-nonenoic acid was based on comparing the retention times of the endophytic volatile and the standard on a GC column. The standard’s retention time (Figure 3A, 14.762 min) and fragmentation (41, 55, 60, 69, 73, 96, 114, 123, 138 *m*/*z*) (Figure 3B) were identical to the endophyte’s product. 

### 2.3. In Vitro 8-Nonenoic Acid Concentration Bioassay

**Figure 4:**
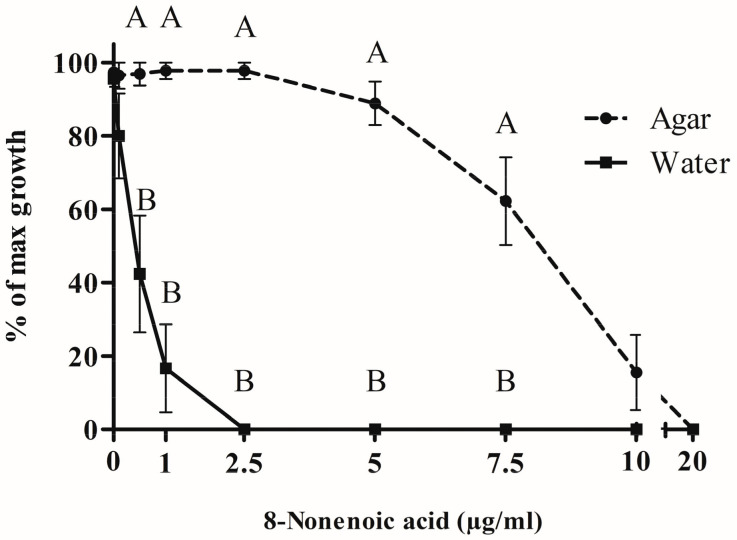
Bioactivity of 8-nonenoic acid against *Saprolegnia parasitica*. Different concentrations of 8-nonenoic acid were added to solid (PDA) and liquid (DDW and PDB) media. Results present the percentage of maximal (control) growth of *S. parasitica* at the different concentrations. The results were subjected to ANOVA followed by Tukey–Kramer multiple comparison test; different letters above points on curve indicate a significant difference between solid and liquid media at *p* ≤ 0.05.

The growth radius of *S. parasitica* mycelium in water + Potato Dextrose Broth (PDB) only (control) was smaller than on agar (5 and 25 mm, respectively). To compare the two types of medium, we calculated the percentage of *S. parasitica* growth compared to the relevant control (Figure 4). No inhibition of *S. parasitica* growth was recorded when 0.1, 0.5, 1 or 2.5 μg/mL 8-nonenoic acid was added in the agar assay, whereas a significant decrease in *S. parasitica* growth was recorded in water for concentrations of 0.5–2.5 μg/mL. Full inhibition was recorded in water at 2.5 μg/mL and above. On the other hand, on agar medium, inhibition of the pathogen was observed only at 5 μg/mL 8-nonenoic acid and above. Reduction of growth on agar continued to full inhibition at 20 μg/mL. We found a significant difference between solid and liquid media for 8-nonenoic acid concentrations of 0.5, 1, 2.5, 5 and 7.5 μg/mL (*p* = 0.0284, 0.0027, <0.001, 0.001 and 0.0064, respectively). In this range, the pathogen was greatly inhibited in liquid media but not, or only barely inhibited in solid media. Viability assays were performed on the fully and partially inhibited *S. parasitica* plugs from both water and solid assays on new media plates. From the solid assay, loss of viability was achieved only with 20 μg/mL 8-nonenoic acid. Loss of viability of plugs from the liquid assay was demonstrated at as low as 0.5 μg/mL 8-nonenoic acid.

### 2.4. In Vivo Influence of 8-Nonenoic Acid on Tilapia Eggs Challenged with S. parasitica 

**Figure 5:**
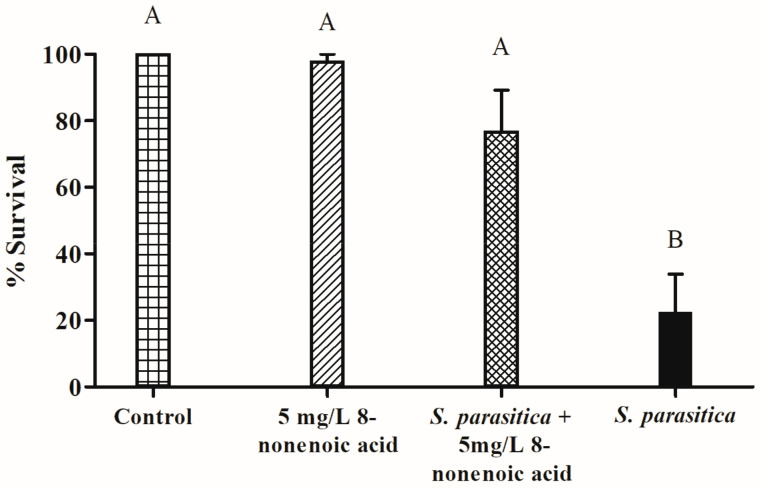
Effect of 8-nonenoic acid on *Saprolegnia parasitica* pathogenicity on tilapia eggs. Percent survival of tilapia eggs 5 days post-inoculation with *S. parasitica* in the presence or absence of 8-nonenoic acid is presented as a column graph. Control: eggs with ethanol only; 8-nonenoic acid 5 mg/L: eggs with 8-nonenoic acid only at a concentration of 5 mg/L; *S. parasitica* + 8-None 5 mg/L: eggs inoculated with *S. parasitica* and 5 mg/L 8-nonenoic acid; *S. parasitica*: eggs with *S. parasitica* only. Results were subjected to ANOVA followed by Tukey–Kramer multiple comparison test; different letters above the bars indicate a significant difference between treatments at *p* ≤ 0.05.

**Figure 6:**
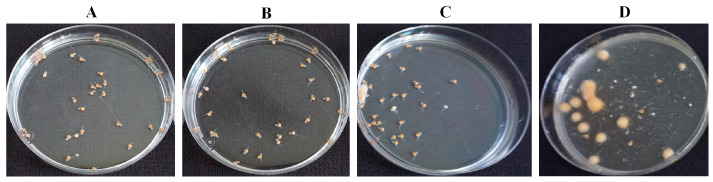
Effect of 8-nonenoic acid on tilapia eggs/larvae inoculated with *Saprolegnia parasitica*, evaluated 5 days post-inoculation. (**A**) Eggs with ethanol only: all of the larvae developed and hatched normally. (**B**) Eggs with 5 mg/L 8-nonenoic acid: 98% of the larvae developed and hatched normally. (**C**) Eggs with 5 mg/L 8-nonenoic acid inoculated with *S. parasitica*: 77% developed and hatched normally, dead eggs did not develop fungal-like growth. (**D**) Eggs inoculated with *S. parasitica*: 22.5% survived, infected eggs developed fungal-like growth.

The survival of tilapia eggs/larvae challenged with *S. parasitica* in the presence of 8-nonenoic acid was examined in in vivo bioassays. We performed a preliminary assay to find the lowest effective concentration of 8-nonenoic acid on *S. parasitica* that we could use in the tilapia egg bioassays. We did not find any additive effect when adding 7.5, 10 or 15 mg/L to the flasks compared to 5 mg/L. We did find a reduction in larvae’s survival when it was treated with 1 mg/L 8-nonenoic acid, a survival rate of 27%. The assays were initiated on eggs that were 4 days postfertilization. At this time point, the eggs have already started to develop but are still a few days before hatching. We evaluated the number of hatched larvae 5 days post-inoculation with the pathogen. The control treatment (eggs with ethanol only) showed 100% survival and proper development of the larvae (Figure 5 and Figure 6A). Within 2 days of adding *S. parasitica* to the flasks, we could see mortality characterized by round filamentous growth around the eggs. Survival rate 5 days post-inoculation was 22.5% (Figure 5 and Figure 6D). In this treatment, all larvae died after 14 days. When 5 mg/L 8-nonenoic acid was added to the eggs challenged with *S. parasitica*, the survival rate increased to 77% (Figure 5). In this case, mortality was not characterized by round filamentous units, but rather by a non-vital egg/larva (Figure 6C). In this treatment, challenged larvae were reared for more than 2 weeks to follow their development, which was normal. Ninety-eight percent of the eggs in flasks that were supplemented with 5 mg/L 8-nonenoic acid only hatched and developed properly, suggesting that the compound could be used in future applications on the eggs at this concentration. No toxicity of 30 mg/L 8-nonenoic acid was observed, and hatching and development were normal. Interactions between treatments were found to be significant: control vs. *S. parasitica* (*p* < 0.001), 5 mg/L 8-nonenoic acid only vs. *S. parasitica* (*p* < 0.001) and 5 mg/L 8-nonenoic acid + *S. parasitica* vs. *S. parasitica* (*p* = 0.0015). 

## 3. Discussion

ABp5 was previously isolated [18] as an endophyte from the seaweed *B. plumosa*. ABp5 demonstrated inhibitory activity against the fish pathogen *S. parasitica* in in vitro assays. We therefore decided to isolate and identify the secondary active molecules that it secretes. Because of the endophyte’s activity in antibiosis petri-plate assays, we initially assumed that the active compounds are secreted into the growth medium. However, our attempts to additionally extract active crude from liquid growth medium were unsuccessful. Yet, when we re-cultured ABp5 from this liquid growth medium on solid agar medium, it was once again found to be active against *S. parasitica*. Therefore, we assumed that the active metabolites are not secreted by the bacteria but are emitted as active volatile compounds. To test this assumption, we conducted an assay designed to evaluate the activity of volatile metabolites by using a two-compartment petri plate. Our results demonstrated inhibition of *S. parasitica* by the volatiles of ABp5 in this assay. 

To identify the VOCs emitted by ABp5, we used SPME. The fiber was incubated with the growing endophyte prior to GC–MS analysis. The GC–MS analysis suggested 10 low-molecular-weight (151–256) molecules from different families of secondary metabolites (Table 1). We purchased nine of them; the volatile compound oxime-, methoxy-phenyl-_ was not available for purchase, but a review of the literature showed that this volatile is known for its anti-viral, anti-bacterial and anti-fungal bioactivity [27,28]. The other nine metabolites were tested in in vitro assays for their inhibitory activity against the aquaculture pathogen *S. parasitica*. Of these, only 8-nonenoic acid exhibited meaningful biological activity against *S. parasitica*. 8-Nonenoic acid is a medium-chain fatty acid with the molecular formula C_9_H_16_O_2_. It is mainly a starting or intermediate material for reactions found in nature [29,30] or in the industry [31,32]. However, it is found as an end product in substances secreted by different animals as a defense mechanism or for behavioral purposes [33,34]. It is also found in extracts of different plants and is considered to have antimicrobial properties [35,36]. 

Although identification of the active bacterial compound from the NIST library was 8-nonenoic acid, we confirmed its identity by two more methods: biological and chemical. The biological method consisted of an antibiosis activity test of the standard against *S. parasitica* (Figure 2). The chemical method consisted of exposing SPME fiber to the 8-nonenoic acid standard and the endophyte under the same conditions and analyzing it by GC–MS. Both methods gave the same result: the active compound in the VOCs and the 8-nonenoic acid standard acted the same (Figure 2 and Figure 3). This, to our understanding, proves that the identity of the emitted compound from ABp5 is 8-nonenoic acid.

A concentration assay in liquid and solid media was performed to determine the most effective concentration for full pathogen inhibition. We were also interested in determining whether the state of the medium (liquid/solid) influences the compound’s activity. Due to the fact that the growth radios of control treatment (*S. parasitica* plug with only water and PDB) in liquid was smaller than control treatment in solid media, we had to normalize the results of *S. parasitica*’s growth radius from all other treatments by presenting the percentage of growth compered to control. This allowed us to compare the efficiency of 8-nonenoic acid’s inhibition between solid and liquid. Since 8-nonenoic acid was discovered as a volatile, we initially tested its ability to inhibit *S. parasitica* as a volatile and not by direct contact (as described in Liarzi et al., 2020). Unexpectedly, 8-nonenoic acid did not inhibit *S. parasitica* in the whole petri-plate area. Therefore, we tested its activity by adding the standard directly into the medium (Figure 4). We think that when this metabolite is added to a solid culture it evaporates to the upper air volume of the compartment. As a volatile, this molecule is active, but the concentration needs to be higher than that in direct contact in order to affect the pathogen. Although the compound’s solubility in water is very limited (284 mg/L at 30 °C) it demonstrates activity at a much lower concentration in water than as a volatile or in solid culture. We do not know why this is the case, but it is not the first time that we have encountered this behavior of volatile metabolites [37]. We speculate it has to do with the molecule ability to move and migrate easier in the liquid media than in the agar matrix but was not demonstrated in our study. 

A simple in vivo bioassay was performed by exposing tilapia eggs to *S. parasitica* in the presence or absence of 8-nonenoic acid. We chose to use tilapia as a model for infected eggs as they are easy to treat, relatively easy to infect with the pathogen and available at the hatchery in the department of fisheries at the Volcani Institute (Rishon LeZion, Israel). We found that 8-nonenoic acid at very low concentrations can control the pathogen and prevent disease development on tilapia eggs, while exhibiting no toxicity to the eggs or larvae, which grew and developed normally. We found a significant effect of the *S. parasitica*-inoculation treatment on the eggs’ mortality compared to the non-inoculated eggs (control) or the eggs treated with 8-nonenoic acid only. This means that the inoculation of *S. parasitica* was successful and that at 5 mg/L, 8-nonenoic acid is not lethal to the eggs. Moreover, addition of 5 mg/L 8-nonenoic acid to eggs infected by *S. parasitica* significantly reduced egg mortality, meaning that it prevents infection of the eggs and enables them to develop normally (Figure 5). As a comparison Willoughby 1992 reported the efficiency of Malachite green oxalate against *S. parasitica* at the 0.25 mg/L in salmonid fish hatcheries [38]. Since 1983, Malachite green oxalate was banned for use in aquaculture and food related applications in many countries due to its classification as a Class II Health Hazard and evidence to its toxicity and carcinogenicity [39]. Other compounds were screened and tested in search after a safe competent control for *Saprolegnia* spp. and other fish pathogens, among those bronopol (2-bromo-2-nitropropane-1,3-diol) was found effective at the ranges of 50–100 mg/L against *S. parasitica* [40], Formalin at the range of 100–400 mg/L, it is needs to be pointed that as with Malachite green formalin is currently not approved as a veterinary medicine for the treatment of live fish in most of the EU countries [39]. Additional compounds known to inhibit *Saprolegnia* spp. are Copper sulphate (1 mg/L), iodophores (50 mg/L) [41], Sodium chloride and a mixture of sodium and calcium chloride (20–30 g/L), Hydrogen peroxide and boric acid (0.2–4 g/L) [39]. Tedesco et al. 2019 described the screening of a list of other relatively considered safe chemicals and found some to be effective at the range of 50–5000 mg/L [39]. our result suggests that 8-nonenoic acid may be used in aquaculture hatcheries to treat the eggs against pathogens, using a low effective concentration. Nevertheless, these results need to be repeated and tested not only under laboratory conditions, but also under hatchery-like conditions. 

Another aspect of this isolate that interested us was its potential pathogenicity to humans. This is relevant to its use as a biocontrol agent in aquaculture facilities. We used the PathogenFinder tool (version 1.1, Elixir bio.tools, Hinxton, Cambridgeshire, UK) [42] on a whole-genome sequence of ABp5, and the isolate was predicted to be non-pathogenic to humans (A1_ PathogenFinder).

To conclude, in this study we demonstrated the biological activity of an algal endophyte against a specific aquaculture pathogen, the oomycete *S. parasitica*. We isolated an active secondary metabolite, identified it in biological and chemical analyses as 8-nonenoic acid, and suggest its use in aquaculture based on the results of in vitro and in vivo bioassays. To the best of our knowledge, this is the first time such a link, and its demonstration between the compound and the pathogen, have been published.

## 4. Materials and Methods

### 4.1. Endophyte Isolation and Maintenance

*K. flava* isolate ABp5 was obtained as an endophyte from the seaweed *Bryopsis plumosa*. The seaweed was sampled from the Mediterranean shoreline, Israel (31°49′03.8″ N 34°38′24.9″ E). The endophyte was isolated as described in Deutsch et al. (2021) [18]. Briefly, the seaweed was surface-sterilized by two washes in 70% ethanol and 5 mm^2^ pieces were placed on nutrient agar (NA) growth medium (Acumedia, Lansing, MI, USA) and incubated at 25 °C for 8 days. The endophytes growing from the seaweed thallus were re-cultivated to obtain a “single-colony” culture. Identity of the isolate was verified using polymerase chain reaction (PCR) amplification and multisequencing analysis as described in Deutsch et al. (2021) [18]. The endophyte was stored in 30% glycerol at −80 °C.

### 4.2. Bioactivity Assays 

Isolate strain ABp5 was grown on NPDA [1/2 NA + 1/2 potato dextrose agar (PDA)] (Acumedia) and incubated at 25 °C for 7 days. Then, the oomycete pathogen *S. parasitica* was introduced to the culture plate by adding a plug of PDA harboring *S. parasitica* mycelia (inoculum from culture of five days growth), on the opposite side of the plate. The culture plate was incubated at 25 °C. Activity was evaluated in a two-compartment petri dish [Figure 1A, for volatile organic compounds (VOCs)], or a one-compartment petri dish (Figure 1B). The effect of ABp5 on *S. parasitica* was examined after 3 days, by comparing *S. parasitica* growth in the presence and absence of the isolate. The pathogen’s viability was evaluated by transferring inoculum plugs from the exposed pathogen to a fresh NPDA plate and evaluating its growth for 7 days (Figure 1C). All assays were performed in triplicate.

### 4.3. VOC Identification

Isolate ABp5 was grown on NAPD agar slant in 20 mL headspace screw-top vials (Thermo Scientific, Langerwehe, Germany). The isolate was spread on the NAPD agar in each vial and incubated at 25 °C for 3 days. A vial with only NAPD agar was incubated as a control, to subtract the media’s volatiles from the sample. A solid-phase microextraction (SPME) fiber assembly with a 50/30 μm polydimethylsiloxane/divinylbenzene/carboxen (PDMS/DVB/CAR) fiber, StableFlex (2 cm) 24 Ga needle, and manual holder (Supelco, Bellefonte, PA, USA) was introduced into the vial headspace for 24 h. The exposed SPME fiber was then inserted into the injector port of a gas chromatography–mass spectrometry (GC–MS) apparatus for 10 min. VOCs were analyzed in a 7890B GC, 5977A GC/MSD system equipped with an HP-5ms (5%-phenyl)-methylpolysiloxane phase capillary column, 1.33 m × 150 μm × 0.25 μm length × diameter × bore (Agilent Technologies, San Diego, CA, USA). The injector temperature was 160 °C, and pulsed splitless injection was used. The detector temperature was 280 °C. The oven temperature was held at 50 °C for 2 min, then increased to 180 °C at a rate of 8 °C/min, and then to 280 °C at 50 °C/min. The recorded mass range was 40 to 800 *m*/*z*, with electron energy of 70 eV. The GC-MS spectrum profiles were analyzed with Mass Hunter software combined with NIST 14 library. The volatiles were identified by comparison of their retention indices with published values (Table 1). 

#### 4.3.1. Testing Standards for Bioactivity against *S. parasitica*

Standards of the compounds identified by GC-MS (Table 1 and Table S1) were purchased and tested for bioactivity against *S. parasitica* by adding 100 μg of each standard dissolved in 100% Ethanol to a sterile 6 mm Whatman filter disk together with plugs of *S. parasitica* mycelia (inoculum from culture of five days growth) on PDA plates at 19 °C (Figure 2). The standard found bioactive against *S. parasitica* (8-nonenoic acid) was subjected to identification by GC comparison. 

#### 4.3.2. 8-Nonenoic Acid Identification by GC Comparison 

Identification of the bioactive compound 8-nonenoic acid was validated by comparing the GC-MS retention time output and molecular fragmentation of the endophytic product with an available authentic standard obtained from Sigma-Aldrich (St. Louis, MO, USA). ABp5 on NPDA agar slant grown for three days and 100 μg/mL of the standard dissolved in double-distilled water (DDW) were exposed to SPME fiber for 24 h. Then, fibers were inserted into GC–MS apparatus and VOCs were compared.

### 4.4. In Vitro 8-Nonenoic Acid Concentration Bioassay

The inhibitory activity of the compound 8-nonenoic acid was examined in both liquid and solid media. For the liquid assay, a 48 well plate was used. A 5 mm plug of PDA harboring *S. parasitica* mycelia (inoculum from culture of five days growth) was introduced into wells containing 1 mL of sterile DDW with 10 μL of potato dextrose broth (PDB) and varying concentrations of 8-nonenoic acid dissolved in ethanol (0, 0.1, 0.5, 1, 2.5, 5, 7.5, 10, 20 μg/mL). The plate was incubated at 19 °C for 3 days. For the solid assay, a 5 mm plug of PDA harboring *S. parasitica* mycelia (inoculum from culture of five days growth) was introduced into 50 mm petri plates with PDA containing varying concentrations of 8-nonenoic acid dissolved in ethanol (as for the liquid assay) and incubated at 19 °C for 3 days. For both liquid and solid assays, mycelium growth radius was measured and percent growth compared to the control (taken as 100%) was calculated. *S. parasitica* plug viability was evaluated by transferring them to fresh PDB/PDA and observing growth for 7 days. All experiments were performed in triplicate.

### 4.5. In Vivo Influence of 8-Nonenoic Acid on Tilapia Eggs Challenged with S. parasitica 

An in vivo bioassay was performed using Mozambique tilapia (*Oreochromis mossambicus*) eggs obtained from the hatchery at the department of fisheries of the Agricultural Research Organization, Volcani Institute. Tilapia eggs, 3 days postfertilization, were collected from the mouth of an adult female and brought to the laboratory. Eggs were rinsed with filtered tap water. Dead, undeveloped and disrupted eggs were removed from the batch. Eggs were counted and then divided into thirty 100 mL Erlenmeyer flasks (10 eggs per flask). The flasks, containing 25 mL filtered tap water, were subjected to five different treatments (two different experiments with three flasks per treatment in each experiment): (1) control treatment—eggs with 12.5 μL ethanol; (2) eggs with 5 mg/L 8-nonenoic acid dissolved in ethanol (12.5 μL ethanol); (3) eggs with 5 mg/L 8-nonenoic acid and two clover seeds inoculated with *S. parasitica*; (4) eggs with 12.5 μL ethanol and two clover seeds inoculated with *S. parasitica*; (5) toxicity treatment—eggs with 30 mg/L of 8-nonenoic acid dissolved in ethanol (12.5 μL ethanol). Flasks were incubated on a shaker incubator at 25 °C, 115 rpm and natural daylight for 5 days. Then the number of viable hatched larvae was recorded.

### 4.6. Statistical Analysis

*S. parasitica* growth (in vitro assay) and larval survival (in vivo assay) values were analyzed with the JMP 10 software package (SAS Inc., Cary, NC, USA). Mean values of % maximal *S. parasitica* growth and % viable larvae were subjected to one-way analysis of variance (ANOVA), followed by Tukey–Kramer multiple comparison test, with significance set to *p* < 0.05.

## Figures and Tables

**Table 1:** Volatiles secreted by isolate ABp5 and identified by GC–MS through a comparison of their retention indices with published values (NIST 14).

Retention Time (Min)	Compound *	Family	Molecular Formula	Mass	Score	Height	Area
6.882	Oxime-, methoxy-phenyl-_	Phenol	C_8_H_9_NO_2_	151.1	79.12	1,321,307	53,113,398
9.488	2-Propyl-1-pentanol	Alcohol	C_8_H_18_O	130.1	85.71	1,055,720	42,679,237
10.773	2-Nonanone	Ketone	C_9_H_18_O	142.1	82.44	2,906,369	85,262,293
12.137	2-Decanone	Ketone	C_10_H_20_O	156.2	75.06	1,167,692	33,491,708
12.383	8-Nonenoic acid	Fatty acid	C_9_H_16_O_2_	156.1	82.06	755,023	28,399,821
14.513	2-Undecanone	Ketone	C_11_H_22_O	170.2	91.03	3,424,025	79,041,922
14.623	2-Undecanol	Alcohol	C_11_H_24_O	172.2	83.82	1,391,479	44,357,455
15.716	2-Dodecanone	Ketone	C_12_H_24_O	184.2	73.23	1,460,936	39,991,523
17.23	2-Tridecanone	Ketone	C_13_H_26_O	198.2	88.75	1,502,874	36,978,277
19.379	2,2,4-Trimethyl-1,3-pentanediol diisobutyrate	Ester	C_16_H_30_O_4_	286.2	83.7	2,495,851	73,829,637

* Identified according to NIST Mass Spectral Library.

## Data Availability

Data is contained within the article or Supplementary Materials. The data presented in this study are available in this article or Supplementary Materials Table S1.

## Supplementary Materials

The following supporting information can be downloaded at: https://www.mdpi.com/article/10.3390/md21090476/s1, Table S1: Volatiles secreted by isolate ABp5–additional information.
