# Supplementary material for: Kocuria flava, a Bacterial Endophyte of the Marine Macroalga Bryopsis plumosa, Emits 8-Nonenoic Acid Which Inhibits the Aquaculture Pathogen Saprolegnia parasitica
Source: Mar Drugs. 2023 Aug 29;21(9):476. doi: 10.3390/md21090476 (PMC10532832; doi:10.3390/md21090476)
Supplement: Supplementary file 1 [file marinedrugs-21-00476-s001.zip › marinedrugs-2530233-supplementary.pdf]

Table S1: Volatiles secreted by isolate ABp5 –additional information.

COMPOUND	SUPPLIER	CAS NUMBER	CHEMICAL STRUCTURE*
Oxime-, methoxy-phenyl-_	-	67160-14-9	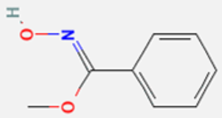
2-Propyl-1-pentanol	Alfa Aesar	58175-57-8	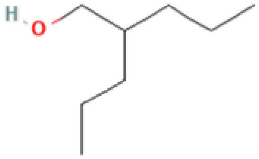
2-Nonanone	Thermo Fisher	821-55-6	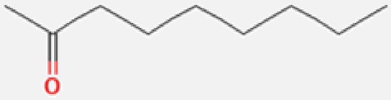
2-Decanone	Alfa Aesar	693-54-9	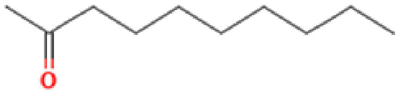
8-Nonenoic acid	Sigma-Aldrich	31642-67-8	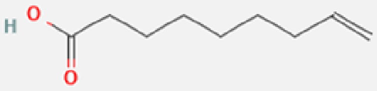
2-Undecanone	Thermo Fisher	112-12-9	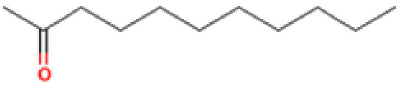
2-Undecanol	Alfa Aesar	1653-30-1	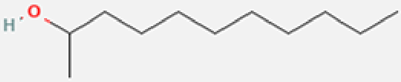
2-Dodecanone	Alfa Aesar	6175-49-1	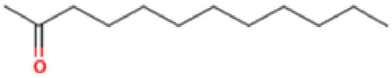
2-Tridecanone	Thermo Fisher	593-08-8	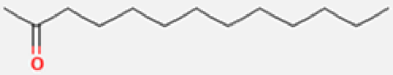
2,2,4-Trimethyl-1,3-pentanediol diisobutyrate	Thermo Fisher	6846-50-0	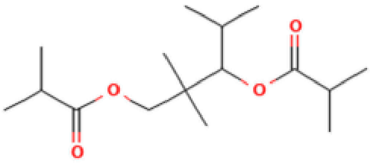

* National Center for Biotechnology Information (2023). PubChem Compound Summary.
